# The Basic Study of Liposome in Temperature-Sensitive Gel at Body Temperature for Treatment of Peritoneal Dissemination

**DOI:** 10.3390/gels8050252

**Published:** 2022-04-20

**Authors:** Ikumi Sugiyama, Kaana Ando, Yasuyuki Sadzuka

**Affiliations:** 1 Department of Advanced Pharmaceutics, School of Pharmacy, Iwate Medical University, 1-1-1 Idaidori, Yahaba-cho, Shiwa-gun 0208-3694, Japan; ysadzuka@iwate-med.ac.jp; 2 Pharmaceutical and Health Sciences, Graduate School of Pharmaceutical Science, Iwate Medical University, 1-1-1 Idaidori, Yahaba-cho, Shiwa-gun 0208-3694, Japan; 2sb.0bu.2py.pick.202@gmail.com

**Keywords:** liposome, temperature-sensitive, gelation, paclitaxel, peritoneal dissemination, antitumor

## Abstract

Peritoneal dissemination is a disease that is difficult to treat surgically because it is widely scattered and proliferates in the abdominal cavity. It is a challenge that even if the drug is administered directly into the abdominal cavity, it rapidly disappears from the abdominal cavity, and the therapeutic effect is not optimal, as expected. In this study, for a liposomal paclitaxel in temperature-sensitive gel that is a suspension before administration and a gel after intraperitoneal administration, the antitumor effect of this formulation was evaluated. Temperature-sensitive gels were prepared using methylcellulose, sodium citrate, and macrogol 4000 and mixed with liposomal paclitaxel. Liposomal paclitaxel containing temperature-sensitive gel in the body was administered into the peritoneal cavity of a mouse model of peritoneal dissemination; the number of cells was significantly reduced compared to a paclitaxel solution of liposomal paclitaxel. These results showed that the liposome in temperature-sensitive gel inhibited cell proliferation in the abdominal cavity. This formulation can be administered easily at room temperature, and it gels and remains in the abdominal cavity for a long period, resulting in a more substantial effect than the existing drug.

## 1. Introduction

Peritoneal dissemination is a particular form of metastasis in ovarian cancer [1], pancreatic cancer [2], and gastrointestinal cancer [3]. The most frequent cause of gastric cancer death is peritoneal metastasis, which is considered an independent prognostic factor for poor prognosis [4]. Peritoneal dissemination is one of the most serious consequences of patients with peritoneal carcinomatosis. The predicted quality of life is very poor and the 5 year survival rate is less than 40% for advanced ovarian cancer and less than 12.5% for colorectal cancers [5,6]. Peritoneal metastasis occurs in a multistep process: (1) detachment of tumor cells from the primary tumor; (2) survival of tumor cell in the microenvironment of the abdominal cavity; (3) attachment of free tumor cells to peritoneal mesothelial cells and invasion into the basement membrane; and (4) tumor growth with the onset of angiogenesis [7,8,9,10]. However, a poor understanding of the molecular mechanisms underlying the peritoneal dissemination process hinders the development of an effective treatment strategy for this condition. 

Removing the peritoneal dissemination is difficult completely, and often recurs by the stranded tumor even after seemingly perfect surgery. Thus, chemotherapy has been the first choice for treatment [11,12,13]; however, chemotherapy for peritoneal dissemination is inadequate due to insufficient drug delivery, and due to symptoms such as intestinal obstruction and abdominal bloating, patients have a poor prognosis [14,15,16]. Intraperitoneal chemotherapy with paclitaxel and cisplatin, in addition to intravenous combination, has attained the most prolonged median survival time in patients with peritoneal carcinomatosis [17,18]. However, such an intraperitoneal approach with taxane and platinum drugs has not yet become a routine practice [19]. Bevacizumab, a monoclonal antibody against vascular endothelial growth factor, has been added to regimens of some combination therapies [20]. Moreover, successful survival improvements with newly developed therapeutic agents, including anti-angiogenic agents and polymeric adenosine diphosphate (ADP) ribose polymerase inhibitors, have been reported [20,21,22]. Thus, one of the most important considerations is to improve the intraperitoneal chemotherapy efficiency. Intraperitoneal chemotherapy transfers high amounts of antitumor agents to the peritoneal site, thereby directly exposing peritoneal neoplasms to high concentrations of these drugs [23]. Intraperitoneal chemotherapy is completed within 30–120 min, which is considered a short time for injection [24], causing insufficient drug delivery to the tumor. In addition, low-molecular-weight drugs are rapidly absorbed by capillaries and enter the circulatory system [25]. 

Liposome, a drug delivery system technology, is used as a carrier to improve drug accumulation in the target site. Liposomes are expected to inhibit the absorption of drugs from capillaries due to their larger molecular weight. Moreover, it has been reported that intraperitoneally administered liposomes with polyethylene glycol is easy to remain in the abdominal cavity than a solution [26]. Rezaeian et al. proposed the intraperitoneal injection of thermosensitive liposomal doxorubicin in combination with mild local hyperthermia induced by high-intensity focused ultrasound [27].

Hydrogels can be prepared with a wide variety of properties, achieving biostable, bioresorbable, and biodegradable polymer matrices, whose mechanical properties and degree of swelling are tailored with a specific application. These unique features give them a promising future in the fields of drug delivery systems and applied biomedicine [28]. In particular, thermo-sensitive hydrogels, which can undergo phase transition or swell/deswell as the ambient temperature changes, endow the drug delivery system with enhanced local drug penetration, desirable spatial and temporal control, and improved drug bioavailability [29]. To date, Oncogel^®^, a thermosensitive poly-lactide-co-glycolic-acid (PLGA)-polyethylene glycol (PEG)-PLGA copolymer-based hydrogel, is the only formulation ever proposed for the local release of paclitaxel in solid tumors, but it brought about no significant improvements in terms of anti-tumor efficacy, so it did not reach the market [30,31].

We hypothesized that liposome in temperature-sensitive gel at body temperature is useful for the treatment of peritoneal dissemination, because the liposomal suspension at room temperature is easily administered, while the gelation liposome at body temperature can remain in the peritoneal cavity and maintain the required drug concentration for a long time. In this study, we carried out a basic investigation of the properties and intraperitoneal retention of the liposome in temperature-sensitive gel to show their potential for the treatment of peritoneal dissemination.

## 2. Results

### 2.1. Characterization of Liposome

The particle size of paclitaxel-loaded liposomes was controlled to 124.0 ± 1.4 nm. The surface zeta potential of the liposomes was −27.2 ± 1.7 mV, because DSPG-Na, a negatively charged lipid, is included as a constituent lipid. Conversely, the potential of a similar formulation without PEG modification was −43.2 ± 3.3 mV. The paclitaxel encapsulated amount was 10.0 ± 3.1 μg/mL. The encapsulated ratio of paclitaxel, a lipophilic drug, into the liposome was 10%. For the encapsulated amount, the ratio of the composition lipids and the presence of PEG modification were examined; the maximum amount of paclitaxel encapsulated was obtained when DSPC: cholesterol: DSPG-Na (molar ratio, 3:6:9) (data not shown). In the following studies, liposomes were prepared in this ratio.

### 2.2. Cytotoxicity of Liposomal Paclitaxel 

**Figure 1:**
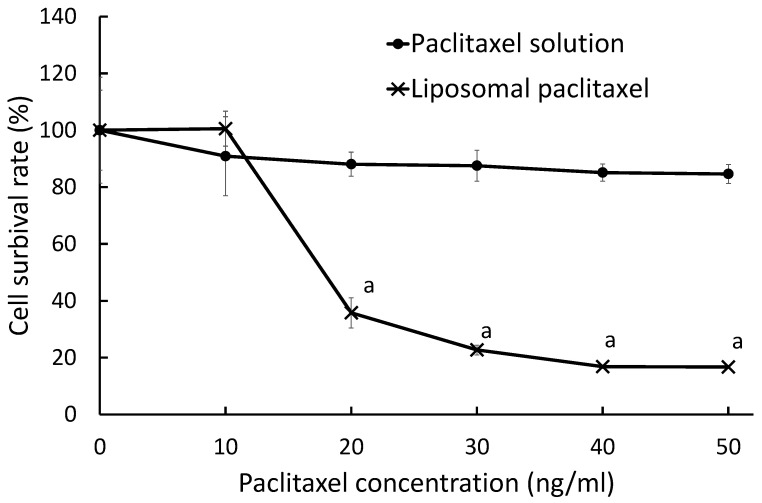
Effect of gelation of paclitaxel on cell viability. P388 leukemia cells (1 × 10^5^ cells/mL) were incubated in a 5% CO_2_ incubator at 37 °C for 24 h, and then paclitaxel solution or liposomal paclitaxel was added. After 48 h of incubation, cell viability was calculated using a colorimetric test using cell counting kit-8. Data are expressed as the mean ± SEM of *n* = 4–8. Significant difference from paclitaxel solution is a: *p* < 0.001.

When the cell suspension was added with paclitaxel solution, the cell survival ratio of 10 and 20 ng/mL of paclitaxel was 90.9 ± 18.6 and 88.0 ± 4.2%, respectively. Even when the concentration of paclitaxel was increased to 50 ng/mL, the cell viability was 84.6 ± 3.4%, namely, there was a minor cytotoxicity enhancement with paclitaxel concentration. Conversely, liposomal paclitaxel improved the cell cytotoxicity as the concentration increased. Although 10 ng/mL of liposomal paclitaxel showed little cytotoxicity, 20 ng/mL of liposomal paclitaxel decreased the cell viability to 35.8 ± 5.3%. When the concentration of paclitaxel was altered from 20 to 50 ng/mL, liposomal paclitaxel displayed a significantly stronger cytotoxicity than its solution. The half maximal inhibitory concentration (IC_50_) of liposomal paclitaxel was 17.1 ng/mL (Figure 1).

### 2.3. Viscosity Changes in Temperature-Sensitive Gels

**Figure 2:**
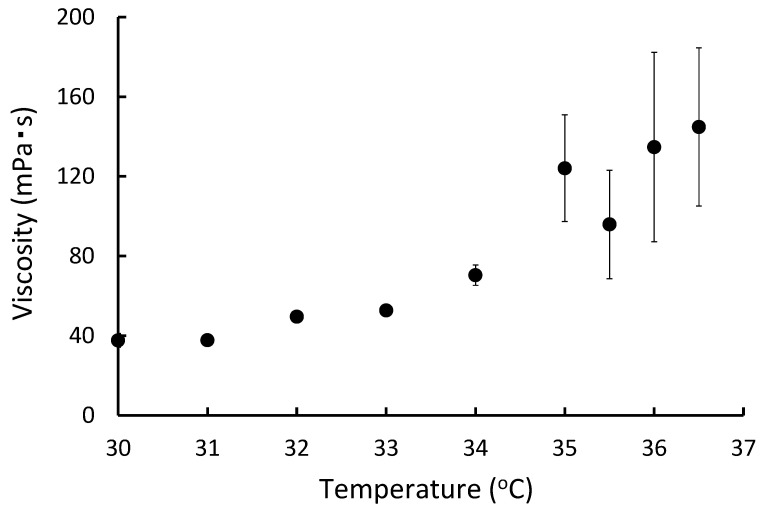
Viscosity evaluation of temperature-sensitive gel against temperature changes. A glass tube was filled with temperature-sensitive gel and placed in a temperature-controlled water bath for 10 min. A 5/32-inch steel ball was dropped from the top, and the time taken for the ball to fall 10 cm was measured. Viscosity was calculated using Stokes’s law. Data are expressed as the mean ± SEM of *n* = 3–5.

The viscosity at 25–36.5 °C was measured to evaluate the temperature sensitivity of paclitaxel in temperature-sensitive gel (PTG). At 25 °C, all gels dropped the steel ball instantly; it was impossible to calculate the viscosity. Alternatively, none of the compositions gelled at 25 °C. As the temperature was increased, the fall time of only paclitaxel containing temperature-sensitive gel D (PTG-D) could be measured when the temperature was above 30 °C, and the viscosity could be calculated. The viscosity increased gradually above 30 °C and tended to increase above 34 °C. The viscosity was 135 mPa·s at 36 °C (Figure 2).

**Figure 3:**
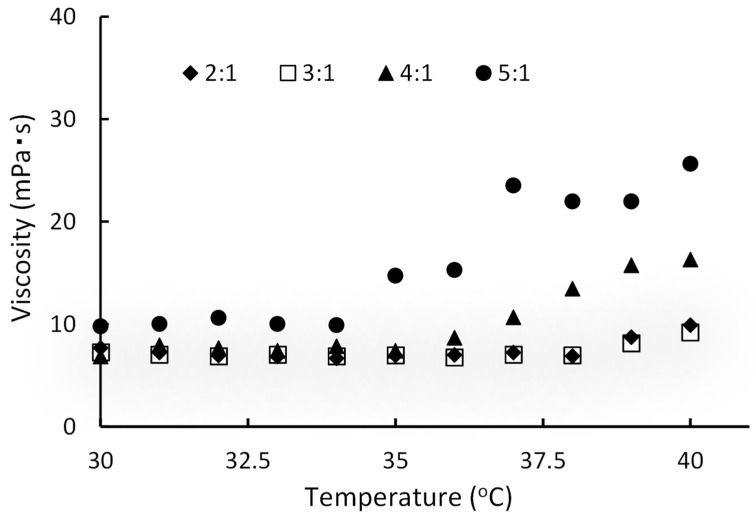
Evaluation of viscosity against temperature change in the mixing ratio of temperature-sensitive gel and liposomal paclitaxel. Viscosities were calculated using Stokes’ law in the same way as the method for measuring the viscosity of the temperature-sensitive gel. The mixing ratios of the gel and liposomal paclitaxel suspension were presented as 2:1, 3:1, 4:1, and 5:1 *v*/*v*.

Next, the alteration in viscosity with the temperature of paclitaxel-contained liposome in temperature-sensitive gel (PLTG) mixed with the temperature-sensitive gel D (without paclitaxel), and liposomal paclitaxel was examined in different ratios. The results revealed that the amount of gel affected the viscosity of the PLTG with increasing temperature (Figure 3). Although the adequate viscosity and gelation temperature can be controlled by varying the mixing ratio of temperature-sensitive gel, and the liposomal paclitaxel, too much temperature-sensitive gel made a hard gel. To make the gel as hard as possible, liposomal paclitaxel resuspended in an equal volume of temperature-sensitive-D was used instead of PBS (pH 7.4) after ultracentrifugation. This formulation, which was resuspended by temperature-sensitive-D, could not measure the fall time at 25 °C, as the steel ball dropped immediately, but at 33 °C or higher, the ball did not fall, as it formed a solid gel (data not shown). Subsequent studies were conducted and found that the mixing ratios of the gel and liposomal paclitaxel suspension was 5:1 *v*/*v*.

### 2.4. Paclitaxel Leak Behavior from Temperature-Sensitive Gels 

**Figure 4:**
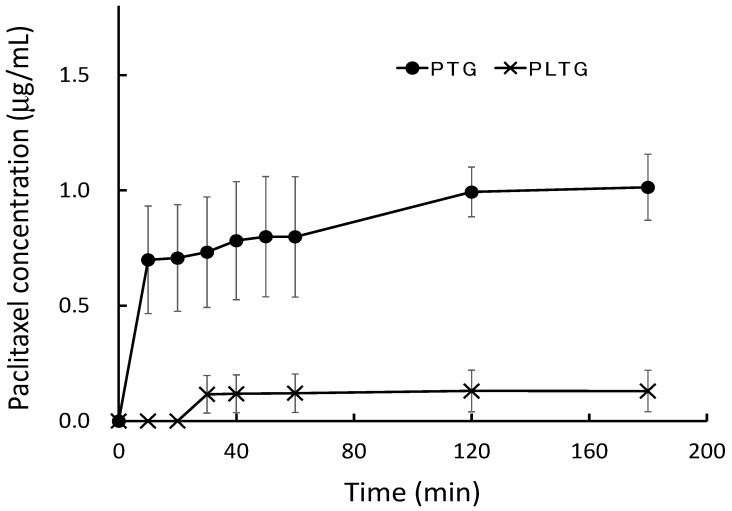
The leak behavior of paclitaxel from the temperature-sensitive gel. The apparatus shown in Section 4.7 was used. These samples were placed on a semipermeable membrane, the receiver solution was sampled at fixed intervals, and the concentration of paclitaxel in the sampled solution was measured using HPLC. The only paclitaxel detected using this method was the free form leaked from these gels of liposomes. Data are expressed as the mean ± SEM of *n* = 3.

To evaluate the leak behavior from the gel formulation, PTG and PLTG were preheated at 37 °C. In PTG, paclitaxel leaked to the receiver solution after 10 min of start. In the first 60 min, 0.8 ± 0.26 μg/mL was leaked, and in the next 60 min, approximately 0.2 μg/mL was leaked. Paclitaxel in PTG leaked 1.20 ± 0.09 μg/mL for 24 h. In the PLTG, paclitaxel could not be detected in the receiver solution until 20 min, and it could be detected after 30 min. Even after 24 h, the paclitaxel concentration in the receiver solution was only 0.33 ± 0.02 μg/mL, which was approximately one-fourth that of the TG group. The release rate of the PLTG was slower than that of the PTG, namely, the paclitaxel leaked more slowly by mixing with temperature-sensitive gel and liposomal paclitaxel (Figure 4).

### 2.5. Antitumor Effect on Peritoneal Dissemination Model Mice

M5076 ovarian sarcoma cells were transplanted into the peritoneal cavity of BDF_1_ mice to make peritoneal dissemination model mice. When M5076 ovarian sarcoma cells were transplanted into the abdominal cavity, the cells proliferated without depositing in any organ, and ascites accumulated in the abdominal cavity. In the preliminary examination, we showed that when M5076 cell suspensions of 1 × 10^6^ cells/mL were transplanted into the abdominal cavity of mice, the cell count reached 1 × 10^7^–1 × 10^8^ cells/mL after 14 days, and the mice died. Therefore, drug administration was started on day 6 of transplantation to account for three repeat administrations.

**Figure 5:**
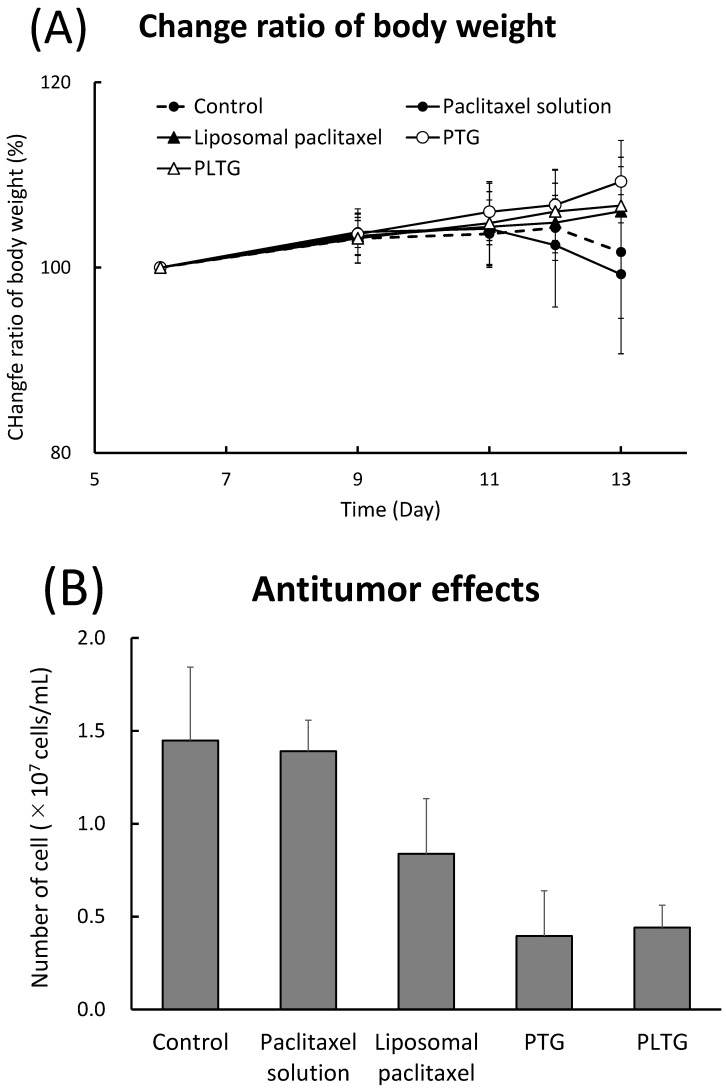
Antitumor effect on a mouse model of peritoneal dissemination. (**A**) Change in body weight. The bodyweight of all mice was measured during the test period. (**B**) The antitumor effect as the number of cells grown in the mouse abdominal cavity. Paclitaxel solution, liposomal paclitaxel, PTG, and PLTG were administered intraperitoneally 6, 9, and 12 days after transplantation of M5076 ovarian sarcoma cells into the abdominal cavity, respectively. At 13 days after transplantation, the cell suspension in the abdominal cavity was collected, and the viable cell count was counted using a hemocytometer. Data are expressed as the mean ± SEM of *n* = 5–7. Significant differences from Control, Paclitaxel solution, and Liposomal paclitaxel are (a) *p* < 0.01, (b) *p* < 0.001, and (c) *p* < 0.05, respectively.

The treatment groups were divided on the basis of body weight as cell proliferation in the abdominal cavity cannot be checked externally regarding the number and size of tumor cells. The average body weight of the control, paclitaxel solution liposomal paclitaxel, PTG, and PLTG was 26.1 ± 1.3, 26.6 ± 1.7, 26.8 ± 1.3, 26.5 ± 1.3, and 26.9 ± 0.6 g, respectively. The control and paclitaxel solution lost body weight from day 11. Other samples remained at a similar level without body weight loss (Figure 5A).

On day 14 of transplantation, cells were collected from the peritoneal cavity and counted. The number of cells in the paclitaxel liposome reduced more than that in the solution. Hence, the liposomalization was useful in improving the anti-peritoneal dissemination effect. PLTG significantly decreased the number of cells compared to the control (*p* < 0.01), paclitaxel solution (*p* < 0.001), and liposomal paclitaxel (*p* < 0.05) (Figure 5B). It was shown that the gelation of the liposome provides a more effective inhibition of cell proliferation by extending the abdominal cavity retention time. When PTG and PLTG were compared, there was no difference between them, and both showed a strong inhibitory effect on tumor proliferation. The protein amount in the collected cell suspension was also measured as the capture data in cell number. There was a correlation between the number of cells and the protein amount (data not shown). The data supported that PTG or PLTG administration strongly inhibited tumor growth.

## 3. Discussion

This study aims to evaluate the usefulness of temperature-sensitive gelation liposomes in treating peritoneal dissemination. Peritoneal dissemination is often induced by gastrointestinal, hepatic, and ovarian cancer. For example, peritoneal dissemination derived from ovarian cancer occurs when cancer cells are distributed into the abdominal cavity due to the failure of the tumor mass surface. Treatment methods for intra-abdominal dissemination include the oral and intravenous administration of anticancer agents. However, these do not provide the expected therapeutic effect, due to the difficulty in transferring drugs into the abdominal cavity. It is also extremely difficult to surgically remove all metastases scattered there. To solve these problems, a novel formulation development focusing on enhancing the retention time in the abdominal cavity was attempted. Two points for developing novel formulations were set: the ability to maintain drugs at a high concentration locally and to pool drugs for a long time in the abdominal cavity, and a liposome designed in the temperature-sensitive gel. This formulation is the liposome suspension that becomes a gel at body temperature after intraperitoneal administration, which is expected to increase abdominal cavity retention and delay the loaded agent release rate, thereby avoiding toxicity to normal cells. Though research has been mainly directed toward macroscopic hydrogels, there is an ever-growing interest in micro and nanogels. Hydrogel nanoparticles are one of the most promising nanoparticulate drug delivery systems owing to their unique nature combining the features and characteristics of a hydrogel system with a nanoparticle [28]. Furthermore, our new focus is that hydrogel with liposomes is thermosensitive.

First, liposomal paclitaxel was prepared to evaluate the liposomalization effect on cytotoxicity. Liposome with a 10.0 ± 3.1 μg/mL paclitaxel content was used and the cytotoxicity at up to 50 ng/mL was examined. The cytotoxic effect of paclitaxel solution was hardly observed within 50 ng/mL, while liposomalization of paclitaxel improved the effect (Figure 1). It was speculated that the enhanced effect of liposomalization is due to the high concentration of paclitaxel released locally. Furthermore, it was suggested that the liposomes were transported into the cells by membrane fusion or endocytosis, thus damaging the cells more efficiently than the solution. This study revealed that liposomal paclitaxel was more useful for enhancing the paclitaxel effect after the leak from the gel than the solution.

The composition of the temperature-sensitive gel, which gels at body temperature after administration, was based on the Rysmon^®^ TG ophthalmic solution [32]. The Rysmon^®^ TG ophthalmic solution contains cellulose, which has reversible sol–gel phase transition properties, so it becomes gelated at body temperature after the eye drop. Furthermore, the addition of citric acid also allows the reversible sol–gel phase transition temperature to be decreased to the body surface temperature. For the formulation that gelled with body temperature, it was possible to alter the gelation and gelation temperature by controlling the mixing ratio of methylcellulose 15, methylcellulose 400, sodium citrate, and macrogol 4000 (Figure 2 and Figure 3). A good increase in viscosity near body temperature was observed when methylcellulose 15, methylcellulose 400, sodium citrate, and macrogol 4000 were mixed at 0.7:0.7:5:2% (*w*/*w*) (Table 1). Therefore, it was thought that a formulation that forms a hard gel at body temperature would be suitable for this study. Thus, PLTG was used, which has a high viscosity near body temperature, and a formulation was chosen in which liposomal paclitaxel was resuspended in the temperature-sensitive gel after ultracentrifugation. The PLTG leak rate was slower than that of the PTG, and the paclitaxel concentration in the receiver solution was 0.33 ± 0.02 μg/mL for 24 h, with this value being about one-quarter of that of PTG (Figure 4). Alternatively, liposomal paclitaxel suspended in temperature-sensitive gel delayed the leak of paclitaxel. This result indicates that paclitaxel in the temperature-sensitive gel disappears from the abdominal cavity after the leak, while the liposomal paclitaxel suspended in the temperature-sensitive gel is expected to remain in there for a longer period. These results also show that the liposomes maintain their figure in the temperature-sensitive gel without bursting.

A disease model in which mouse M5076 ovarian sarcoma cells were transplanted intraperitoneally was used to investigate the antitumor PLTG effect. When M5076 ovarian sarcoma cells were transplanted into the abdominal cavity, the cells proliferated without depositing in any organ, and ascites accumulated in the abdominal cavity. The first dose of PLTG was administered 6 days after the cells were transplanted. Preliminary studies have shown that most mice die after 14 days of intraperitoneal transplantation of M5076 ovarian sarcoma cells. Therefore, the administration schedule was designed to allow completion of three doses once every three days by 13 days. We confirmed that cells were still growing in the abdominal cavity on day 6. Starting from the 6th day of transplantation and when cells were collected from the intraperitoneal cavity on the 13th day, the number of cells differed significantly among the treatment groups (Figure 5). The number of cells in the control and paclitaxel solution was comparable, and no antitumor effect was observed with the solution. This is because the paclitaxel solution administered intraperitoneally is quickly transferred into the bloodstream via the capillaries and lymphatics of each organ [33]. In the liposomal paclitaxel, the number of cells was decreased to approximately 58% of the control level. Unlike the paclitaxel solution, it was believed that liposomes with a particle size of approximately 100 nm are less likely to migrate to lymphatic vessels or be absorbed from organ surfaces, resulting in an increased retention period in the abdominal cavity. The antitumor effects of the temperature-sensitive gel, PTG and PLTG, were significantly stronger than the control (*p* < 0.01) and liposomal paclitaxel (*p* < 0.05). We chose a method using a hemocytometer to show the antitumor effect to obtain basic data in a simplified manner. Essentially, cell counting by FACs would be an appropriate method to determine the antitumor effect accurately. In this study, as we were able to demonstrate that the liposome in temperature-sensitive gels was useful for treatment of peritoneal dissemination as a basic study, we evaluate the effect and mechanisms in more detail in the next step. 

It was considered that the paclitaxel gel at body temperature increased the retention time and provided a sustained leak effect in the abdominal cavity. However, there was no difference in the antitumor effect between PTG and PLTG. As shown in Figure 4, the release of paclitaxel from PLTG was extremely slow, and the paclitaxel amount leaked even after 24 h was extremely small, so it was speculated that one of the causes may be that the gel was too hard in the abdominal cavity. Conversely, PLTG had a similar level as PTG on cell proliferation, even though the amount of paclitaxel leakage was lower. This result is associated with the finding that the liposomal paclitaxel was more cytotoxic (Figure 1), and it was expected that the liposomes directly approached the tumor cells after leaving the gel and achieved a similar level with a smaller amount. In the future, an opportunity to investigate in detail and clarify whether the firmness of the gel affects the antitumor effect and leakage of paclitaxel would be appreciated. There is one more major difference between PLTG and PTG. PLTG has a lower amount of free paclitaxel in the abdominal cavity. This is a significant advantage regarding the reducing side-effects. In cancer chemotherapy, it is imperative to develop a formulation with fewer side-effects to continue the treatment. It was hoped that PLTG administration is helpful in terms of side-effects, and there are plans to research more in the future. The sustained release capability of PLTG is also an advantage in the treatment. PTG and PLTG were studied on the same schedule in this study, but a similar or better effect might have been obtained with a longer interval of PLTG administration. This is a very significant benefit in reducing patient burden.

These results indicate that the PTG can be easily administered as a liquid, and it gels at body temperature and remains in the abdominal cavity for a long period, and then gradually releases paclitaxel. It was suggested that PLTG, a suspension of liposomal paclitaxel in a temperature-sensitive gel, tended to produce an even more efficient increase in antitumor effect. PLTG will be expected to become an unprecedented therapeutic formulation for peritoneal dissemination by adjusting the gel firmness in detail.

## 4. Materials and Methods

### 4.1. Materials

1,2-Distearoyl-sn-glycero-3-phosphocholin (DSPC; COATSOME MC-8080), 1,2-distearoyl-sn-glycero-3-phosphoglycerol, sodium salt (DSPG-Na; COATSOME MG-8080LS), and 1,2-distearoyl-rac-glycero-3-methylpolyoxyethylene, for which the PEG molecular weight is 2000 (PEG2000-DSG; SUNBRIGHT GS-020), were gifts from NOF Co., Ltd. (Tokyo, Japan). Paclitaxel, cholesterol, sucrose, lactic acid, sodium lactate, chloroform, and methanol were purchased from FUJIFILM Wako Pure Chemical Corp. (Osaka, Japan). Methyl cellulose 15, methyl cellulose 400, sodium citrate hydrate, and macrogol 4000 were also purchased from FUJIFILM Wako Pure Chemical Corp. (Osaka, Japan). Cell counting kit-8 was purchased from Dojindo Laboratories (Kumamoto, Japan). RPMI-1640 medium “*Nissui*” was obtained from Nissui Pharmaceutical Co., Ltd. (Tokyo, Japan). In addition, 2-mercaptoethanol was purchased from Nakalai Tesque (Kyoto, Japan). All other chemicals were commercially obtained products of reagent grade.

### 4.2. Animals

The Institutional Animal Care and Use Committee of Iwate Medical University approved the animal experiment. Male BDF_1_ mice (6 weeks old, 21–26 g) were purchased from Japan SLC Inc. (Hamamatsu, Japan). The animals were given a standard experimental diet and water and maintained under controlled temperature (23 ± 1 °C) and humidity (55 ± 5%).

### 4.3. Preparation and Characterization of Paclitaxel-Loaded Liposomes

Liposomes were composed of DSPC: cholesterol: DSPG-Na (molar ratio, 3:6:9) and added with 5.9 mM PEG2000-DSG as modifying material around the liposomes. Paclitaxel was also added with these components. These materials were dissolved in chloroform/methanol (4:1, *v*/*v*), and the organic phase was removed using a rotary evaporator. The formed thin lipid was added to 9% sucrose in 10 mM lactate buffer (pH 4.0) for hydrate and sonicated for 20 min. Then, the liposomes were adjusted to particle sizes of 100 –200 nm using an extruder. The liposomes were dialyzed in 9.0% sucrose in 10 mM lactate buffer (pH 4.0) at 4 °C for 16 h to remove unloaded paclitaxel.

Particle sizes and the zeta potential were determined using a Zeta Sizer Nona-ZS (Malvern Instruments Ltd., Malvern, UK). The liposomes were dispersed in 9% sucrose in 10 mM lactate buffer (pH 4.0), and the average value measured four times was the particle size and zeta potentials. The amount of paclitaxel loaded into the liposomes was measured using a high-performance liquid chromatography (HPLC) system equipped with a UV-VIS detector (Shimadzu SPD-10AVvp, Shimadzu Co., Kyoto, Japan) using water-acetonitrile (40:60, *v*/*v*). The TSKgel ODS-100V (3 μm, 1.0 nm ID × 15.0 cm) (Tosoh Bioscience, Inc., Tokyo, Japan) column had a flow rate of 1.0 mL/min and was maintained at 25 °C. The detector wavelength was set at 230 nm. Peaks were assigned by column elution with analytical standards, and quantitation was performed using standard curves prepared from an analytical standard.

### 4.4. Cytotoxicity

P388 mice leukemia cells (1 × 10^5^ cells/mL) suspension in RPMI1640 medium were seeded in a 96-well plate and incubated in a 5.0% CO_2_ incubator at 37 °C for 24 h. Then, these cells were added to paclitaxel (10–50 ng/mL) and incubated for 48 h. A similar-concentration paclitaxel solution was seeded in 1% DMSO as control. After 48 h, cell counting kit-8 was seeded into the cell samples and reacted in the 5.0% CO_2_ incubator. The cell survival ratio was calculated by measuring the absorption of reaction samples using a microplate reader (λ = 420 nm) [34].

### 4.5. Preparation of Temperature-Sensitive Gel

The temperature-sensitive gel was prepared by reference to the composition and rate of the Rysmon^®^ TG ophthalmic solution (Kissei Pharmaceutical Co., Ltd., Nagano, Japan) [28]. Methylcellulose 15, methylcellulose 400, sodium citrate, and macrogol 4000 were completely dissolved in water in the mass ratio (*w*/*w*%) shown in Table 1 (gels A–D). PTG was dissolved in this solution to a concentration of 1 mg/mL.

PLTG was prepared by mixing liposomal paclitaxel and temperature-sensitive gel. First, the paclitaxel liposomes were treated in an ultracentrifuge (70,500× *g*, 2 h, 4 °C), and the supernatant was removed. The pellets resuspended in temperature-sensitive gel or mixed with temperature-sensitive gel at different ratios after resuspending the pellets in PBS (pH 7.4) were used as PLTG.

### 4.6. The Viscosity of Temperature-Sensitive Gels

A glass tube (φ = 8 mm) was filled with TG and placed in a temperature-controlled water bath. After 10 min, a 5/32-inch steel ball was dropped from the top of the glass tube, and the time to move 10 cm in the TG was measured. Viscosity was calculated from the translation time using Stokes’ law.

### 4.7. Paclitaxel Leak from PTG and PLTG

**Figure 6:**
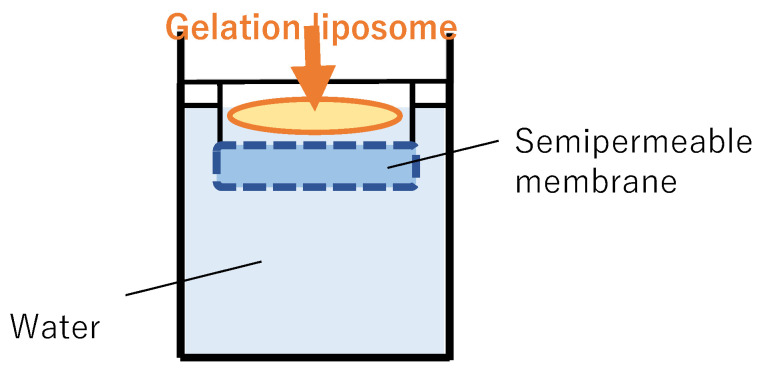
The device of free paclitaxel leak test. A tube with a semipermeable membrane attached to the end was prepared, and PTG or PLTG, which was pre-heated at 37 °C and gelated, was placed on the semipermeable membrane. The receiver solution was water heated to 37 °C. The temperature of the receiver solution was maintained at 37 °C and stirred during the test. The receiver solution was sampled over time.

The apparatus shown in Figure 6 was used to evaluate paclitaxel leak from PTG. The receiver solution was water at 37 °C. PTG and PLTG were preheated at 37 °C and gelled, and they were then placed on a semipermeable membrane. After that, the receiver solution was sampled at fixed intervals and the amount of leaked paclitaxel from PTG or PLTG was measured. Paclitaxel concentration was determined by HPLC using the method described in Section 2.3. As liposomes in PLTG cannot penetrate the semipermeable membrane, only free paclitaxel leaked from liposomes or gels can be detected using this method.

### 4.8. Peritoneal Dissemination Treatment Study (In Vivo)

Peritoneal dissemination model mice were taken by intraperitoneal transplantation of M5076 mouse ovarian sarcoma cells (1 × 10^6^ cells/animal) in BDF_1_ mice (male, 6 weeks old). After 6 days, each mouse was divided into five groups; control (n = 5), paclitaxel solution (n = 7), liposomal paclitaxel (n = 7), PTG (n = 7), and PLTG (n = 7). At 6, 9, and 12 days after transplantation, each sample was intraperitoneally administered at a dose of 50 μg/kg of paclitaxel. The body weight of mice was measured during the test period. Twenty-four hours after the last administration, 5.0 mL of saline was injected into the abdominal cavity, and the tumor cells were removed by collecting the cell suspension. The number of tumor cells in the suspension was counted using a hemocytometer. Additionally, the protein amount of the collected tumor cell suspension was also measured. The amount of protein amount in the cell suspension was determined using the Lowry method [35,36]. The collected cell suspension was centrifuged at 150× *g* for 3 min, the supernatant was removed, and the cell pellets were washed thrice with PBS (pH 7.4). The cells were suspended 1000-fold in PBS (pH 7.4), the reagent was added for protein coloration, and it was left to stand for 10 min at room temperature. Next, Folin–Ciocalteu phenol reagent was added and incubated at 50 °C for 10 min, after which the samples were left to stand at room temperature. The resulting samples determined the absorption (λ = 750 nm) and the protein concentration calculated from the standard curve.

### 4.9. Statistical Analysis

Data were expressed as the mean and standard error of the mean of multiple determinations. The significance of the differences in the mean values of two groups using the Student’s unpaired *t*-test was evaluated. Statistical analyses were performed using IBM SPSS Statistics Version 22 (IBM Corp., Armonk, NY, USA).

## Figures and Tables

**Table 1:** Composition of temperature-sensitive gel.

	Methylcellulose 15 (%(*w*/*w*))	Methylcellulose 400 (%(*w*/*w*))	Sodium Citrate (%(*w*/*w*))	Macrogol 4000 (%(*w*/*w*))
A	0.35	0.35	1.75	1.00
B	0.70	0.70	3.50	2.00
C	0.525	0.525	8.75	1.50
D	0.70	0.70	5.00	2.00

## Data Availability

Not applicable.
